# Legal document summarization: a short review

**DOI:** 10.3389/frai.2026.1787315

**Published:** 2026-06-18

**Authors:** Muaadh Nazly, Prasan Yapa

**Affiliations:** 1 School of Computing, Robert Gordon University, Aberdeen, United Kingdom; 2 Kyoto University of Advanced Science (KUAS), Kyoto, Japan

**Keywords:** argumentation mining, citations and precedents, legal judgment summarization, natural language process (NLP), rhetorical structure

## Abstract

This mini review surveys published work on automatic summarization of legal judgments. We focus on how natural language processing handles argument structure, discourse, and the role of citations and precedent when systems generate shorter versions of a case. The field began with extractive methods and classical machine learning, and moved through graph-based and neural models, and to recent transformer architectures and large language models (LLMs). Some of the recent papers pair LLMs with retrieval augmentation so that outputs are faithful to the source text. A scheme is repeated in the domain that whether researchers should work at sentence or clause level, how rhetorical roles are defined, and how to judge a summary when fluent wording can still misrepresent the judgment. Many limitations still exist where many studies rely on general-domain setups or small, jurisdiction-specific corpora; citations are often treated as meta-data rather than part of the argument; and evaluation still depends heavily on overlap with reference summaries, which only partly reflects legal quality. It is also non-practical to compare results when datasets and benchmarks differ from one country to another. We situate this review next to wider surveys of legal summarization and with LLMs in law, but with only the judgment documents. Finally, we guide in future directions with more languages and jurisdictions, summaries that users can trace back to the judgment, and evaluation that mixes automatic scores with expert review where it is practical.

## 1 Introduction

Legal judgments are long and complex documents. They bind together facts, issues, analysis, and conclusions, usually through narrations of arguments and frequent citations to earlier cases. Summarizing them without losing legally important reasoning is difficult. As a result, automatic legal summarization has grown into a unique research area. This review traces how the research area has evolved, from early feature-based and thematic-segmentation approaches to neural and transformer-based models, and to recent work with LLMs, while preserving the argument structure and precedent.

Several motivations guide the synthesis of this review. Although legal document summarization has advanced, several gaps remain across methods. For example, many summarization pipelines still prioritize sentence-level units and underuse clause-level argumentation. Precedent citations are often treated as peripheral rather than as part of the reasoning structure. These limitations matter for both research design and for any eventual use in legal practice.

The review is organized around the following questions: (1) How has automated legal summarization evolved over time? (2) How far do current systems combine argumentation-oriented structure with precedent or citation information? (3) What gaps remain in summarization frameworks? (4) How might future work better connect theory (discourse, argumentation) with computational implementations? These questions structure the discussion below.

### 1.1 Search and selection

We gathered articles from 2004 to 2025 via Google Scholar, IEEE Xplore, the ACL Anthology, and arXiv using keywords such as “legal document summarization”, “judgment summarization”, “argumentation mining”, “precedent citation”, “legal NLP”, and “large language model legal”. About 150 sources were collected at the initial stage. After screening for relevance to the questions above, methodological contribution, and coverage of recent developments, the review focuses on a core set of about 40 papers, plus survey articles for context. The synthesis relies exclusively on published, citable scholarly sources.

### 1.2 Positioning

General surveys already map legal summarization or LLM applications in law (Jain et al., 2021; Akter et al., 2026; Siino et al., 2025; Dehghani et al., 2025; Siino, 2025). Our aim here is narrower: we emphasize *judgment-oriented* summarization and the relationships between rhetorical-argumentative segmentation, citation and precedent awareness, and evaluation practice. Rather than duplicating the exhaustive bibliometric coverage found in those surveys, we use their findings to establish a framework for the current mini-review.

The remainder of the paper is structured as follows. Section 2 presents theoretical and conceptual background. Section 3 reviews methodologies, including preprocessing and evaluation. Section 4 states key research gaps. Section 5 discusses implications of the reviewed work, and Section 6 concludes.

## 2 Theoretical and conceptual background

Before exploring methodologies, it is useful to clarify concepts that builds the foundation for automated summarization of legal texts. Legal documents are challenging to read and to model since they are long, genre-specific, and rich in cross-references. Work in this area typically intersects at NLP, argumentation theory, and rhetorical structure analysis.

### 2.1 Nature of legal texts and the requirement for summarization

Judicial decisions are not mere narrative descriptions; they form a distinct genre with argumentative, hierarchical, and referential structure (Ashley, 2017). Typical elements in a judgment such as facts, issues, analysis, and disposition play distinct rhetorical parts in the court's reasoning. Manual reading is time-consuming when lawyers or judges must compare many cases or trace lines of authority (Takale, 2023). Automated summarization is therefore motivated by the need for faster access to information, provided that outputs remain faithful to the source judgment.

Early work often relied on extractive summarization by selecting important sentences. Such methods improved accessibility but frequently did not model the argumentative organization of the judgment in depth. That limitation pushed the field toward representations that capture discourse and argument links more explicitly (Kanapala et al., 2019).

### 2.2 Foundations of natural language processing in the legal domain

NLP involves the computational processing of natural language, covering both linguistic analysis and the advanced text generation capabilities of modern frameworks. Historically, the field moved from rule-based and statistical approaches toward neural models. The transformer architecture (Vaswani et al., 2017) underpins the encoder-decoder and encoder-only pretrained models. BERT is a widely used encoder-only model built on top of the transformer stack (Devlin et al., 2019), which was later adapted into the legal domain.

Standard NLP components like tokenization, syntactic parsing, & semantic encoders support legal applications such as retrieval, citation analysis, and summarization (Zhong et al., 2020). Legal language differs uniquely from general news or web text in vocabulary, syntax, and convention (Jayakumar et al., 2023). Domain-adapted models such as LegalBERT aim to reflect legal corpora and long documents more faithfully (Chalkidis et al., 2021). It should be noted that even when summarization is categorized as “sentence-level”, the underlying models continue to operate on subword tokens. The distinction lies in the architectural unit used for composition rather than the absence of tokenization (Xu and Ashley, 2023; Nigam et al., 2025).

### 2.3 Argumentation theory

Argumentation theory describes how conclusions are supported by premises and how counter-arguments are handled. In judgments, such structure often appears through rhetorical roles and cited authority. Identifying roles supports interpretation beyond isolated sentence ranking.

Computational research links classical argumentation frameworks such as Toulmin's claims and warrants, Walton's dialogue schemes, to the NLP tasks such as segment classification and relation prediction (Bench-Capon and Dunne, 2007). These foundations justify pipelines that extract and label premises, conclusions, or contextual spans prior to summarization.

#### 2.3.1 Neural operationalizations

In modern Transformer-based architectures, argumentation schemes are rarely implemented as explicit symbolic inference rules. Instead, the field has moved toward a more fluid approach, where rhetorical or argumentative roles are assigned to sentences or tokens. In this approach, models are trained using standard classification objectives and contextualized representations from Transformer encoders (Poudyal et al., 2020; Nigam et al., 2025). Attention layers may provide learned alignments, but these should not be read as a faithful implementation of formal argumentation theory. Classical schemes like Toulmin's model guide annotation design and error analysis rather than being explicitly encoded in attention heads or loss functions (Bench-Capon and Dunne, 2007).

### 2.4 Rhetorical Structure Theory (RST)

Rhetorical Structure Theory (RST) conceptualizes text as a hierarchy of discourse relations among distinct segments (Taboada and Mann, 2006). The framework pivots on the distinction between nuclei, which are segments essential to the primary communicative goal, and satellites, which provide secondary material like background or evidence. For summarization, this hierarchy is critical where nuclei typically represent the core content required for a concise headnote, while satellites offer candidates for compression or omission, depending on the required level of fidelity.

In legal NLP, RST-related ideas align with labeling rhetorical roles such as facts, arguments, and conclusions in judgments (Malik et al., 2022), which in turn can guide extractive or abstractive summarization.

### 2.5 Conceptual foundations of legal summarization

Legal summarization aims not only to shorten text but to preserve reasoning structure. A useful summary should reflect how the court moved from facts to holding (Shukla et al., 2022). Many pipelines decompose the task into segmentation, representation of units, and ranking or generation (Huang et al., 2021).

Extractive methods copy source spans whereas abstractive methods generate new wording, often with transformer-based decoders (Shukla et al., 2022). Abstractive systems can score higher on ROUGE or BLEU in some settings yet remain vulnerable to hallucination, fragmentation, or loss of legal nuance (Deroy et al., 2023). Mitigations discussed in recent work include retrieval-augmented generation, explicit grounding to citations, and human review for high-stakes use (Deroy et al., 2023; Dehghani et al., 2025).

### 2.6 Toward a theoretical integration

Integrating AI with argumentation schemes supports treating judgments as structured networks rather than flat sequences of sentences (Nigam et al., 2025). This review follows that thread by emphasizing how summarization research engages with the rhetorical organization of court rulings and with the balance between jurisdiction-specific nuance and general NLP benchmarks.

## 3 Review of related studies and methodologies

**Figure 1:**
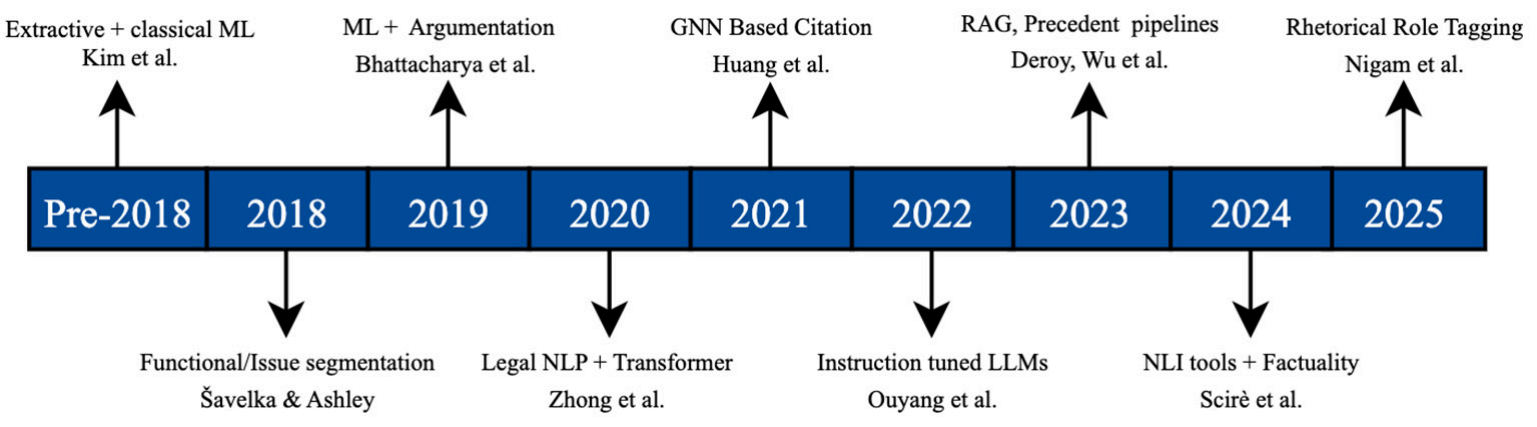
Evolution of automated legal summarization.

Legal document summarization has evolved over around two decades, from extractive and rhetorical segmentation toward argument mining, citation-aware modeling, and LLM-based generation. This section summarizes representative lines of work and their limitations (Figure 1).

### 3.1 Early stage of legal summarization

Early extractive systems used lexical and positional features and machine learning classifiers. For example, support vector machines and random forests to rank candidate sentences (Kanapala et al., 2019). Graph-based extractive summarization, specifically those incorporating cohesion constraints, represents a significant pre-Transformer research line (Kim et al., 2013). These methods established strong baselines but often treated sentences as loosely independent, which limits modeling of argument flow across the judgment.

### 3.2 Transformer-based and deep learning methods

Deep learning brought contextual encoders and neural summarizers. BERT-style models and their legal adaptations such as LegalBERT improved modeling of long, specialized texts (Chalkidis et al., 2021). Studies on Indian Supreme Court judgments indicate that while Transformer-based abstractive models significantly outperform classical baselines in fluency and semantic overlap, they are not without trade-offs. Despite these gains, such models still struggle with factual consistency and structural integrity, occasionally generating summaries that deviate from the legal logic of the original text (Shukla et al., 2022; Deroy et al., 2023). Hierarchical attention and long-context architectures address length but do not automatically resolve clause-level dependencies or citation-heavy passages (Zhong et al., 2020). Tasks like citation recommendation illustrate how metadata-driven neural rankers can sense the importance of specific text spans (Huang et al., 2021). In the legal domain, these same contextual signals provide a roadmap for summarization, informing the model's decision on which parts of a judgment are essential for a concise headnote.

### 3.3 Large language models, multilingual settings, and grounding

A prominent recent trend in legal AI involves the transition to LLM-based frameworks for processing judicial text. The wider move toward instruction-following and human-aligned fine-tuning of large models is grounded in general-domain work on learning from human feedback (Ouyang et al., 2022). Recent surveys highlight how these models are being adapted for core functions, ranging from automated summarization and document classification to sophisticated RAG-based systems (Siino et al., 2025; Dehghani et al., 2025). Experimental results in judicial summarization warn that although high overlap scores are impressive, they can often hide critical legal inaccuracies, forcing the use of robust verification layers (Deroy et al., 2023). Furthermore, comparing general-purpose LLMs with smaller, specialized models indicates that parameter scale is no substitute for domain adaptation and the nuances of legal reasoning still require targeted tuning (Jayakumar et al., 2023).

A summary can read well but still be legally wrong. For example, if it changes what the court decided, drops an important condition, or points to a case that does not support the claim, then it misleads. LLM-based summarizers remain vulnerable to such errors. Recent work therefore adds verification layers in practice: outputs are tied to the source judgment, statements and citations are checked against that text, and tools flag invented or mismatched precedents. For high-stakes use, human oversight is not optional in the sense used across this literature; a qualified reader must still confirm the summary, because the model alone cannot guarantee that the law is stated correctly (Deroy et al., 2023; Dehghani et al., 2025; Magesh et al., 2025).

#### 3.3.1 Multilingual evidence

Legal language and headnote styles vary significantly by region, so multilingual evidence is essential for a complete view of the field. A dedicated survey of LLM use in the Italian legal domain (Siino, 2025) complements applied studies on Italian legal news summarization and on retrieval resources for the Italian Civil Code built with LLMs (Benedetto et al., 2025; Proietti et al., 2025). Together, these sources check the assumption that English-language datasets are a universal proxy for all legal reasoning (Chalkidis et al., 2019; Malik et al., 2022).

### 3.4 Semantic segmentation and argumentation mining

Choosing the right unit of segmentation whether sentences or finer clauses is a critical design decision in legal NLP. Although sentence-level tagging is a common practice, it often overlooks the multiple propositions nested within complex legal phrasing, whereas finer-grained analysis can more accurately map a judgment's logical flow (Xu and Ashley, 2023).

In many pipelines, “sentence-level” means that salience rankers, extractive selectors, or rhetorical-role classifiers treat each sentence as one decision unit. A single judicial sentence can still bundle several legally distinct commitments, for example, a general holding, a limiting exception, and a remedy or disposition expressed together. If summarization only scores or selects whole sentences, compression may preserve fluency while silently merging, dropping, or misaligning one of those embedded commitments. Clause-level, span-level, or proposition-level units therefore change what can be ranked, paraphrased, or tied to citations independently; they are a design choice about argumentative fidelity, not only a preprocessing detail (Xu and Ashley, 2023; Nigam et al., 2025).

These structured inputs, extracted from rhetorical and argumentative classification, are key to improving summary quality (Poudyal et al., 2020; Nigam et al., 2025). Nevertheless, high annotation costs and the persistent ambiguity of functional roles across different legal domains remain significant practical challenges.

### 3.5 Graph-based representations and Graph Neural Networks (GNNs)

By modeling legal text as a graph, researchers can treat sentences, spans, or entire cases as nodes, with edges representing the connective tissue of similarity, discourse relations, or formal citations (Kim et al., 2013; Yasunaga et al., 2017). This relational view is particularly powerful when paired with message-passing Graph Neural Networks (GNNs), which iteratively refine node embeddings by aggregating information from their local neighborhood. The use of directed edges allows models to distinguish between citing and cited units, which is a distinction that is fundamental to the hierarchical structure of legal precedent (Yasunaga et al., 2017; Zhao et al., 2022). Architectural variations from local convolutions to learned attentions, they all share a common vulnerability. They rely on the graph being a faithful representation of legal logic. If the graph is noisy, the summarization output will likely be erroneous. Furthermore, as we move toward summarizing longer, more complex judgments, the field faces a dual challenge where managing the computational costs and ensuring that the foundational steps of segmentation and citation extraction are accurate enough to support the model's reasoning (Huang et al., 2021). Recent judgment summarization work explicitly integrates semantic encoders with structural signals derived from case-specific graphs (Dan et al., 2025), and citation graphs support extractive summarization of key rulings (Bindal et al., 2023). Frameworks that combine domain models with LLMs over retrieved precedents illustrate how inter-document material can enter downstream legal tasks (Wu et al., 2023).

### 3.6 Pre-processing and evaluation

Summarization quality depends on preprocessing stages. Sentence splitting, normalization, and named entity recognition affect downstream argument analysis (Chalkidis et al., 2019; Jurafsky and Martin, 2008; Xu and Ashley, 2023; Kalamkar et al., 2022). Legal-specific segmentation and rhetorical-role labeling are still unevenly supported compared with general-domain tools (Bhattacharya et al., 2019; Shukla et al., 2022).

#### 3.6.1 Evaluation metrics and protocols

Evaluation of text summarization often relies on ROUGE, BLEU, or classification on F1 (Lin, 2004; Papineni et al., 2002). These measures capture lexical overlap but align weakly with argumentative coherence or legal correctness (Šavelka and Ashley, 2018). Studies with legal experts highlight interpretability and consistency criteria that automated metrics tend to miss (Ghosh et al., 2022). Dedicated legal summarization benchmarks help standardize tasks and baselines (T.y.s.s., Santosh et al., 2024). Moving beyond simple lexical overlap, recent general-domain research has pivoted toward Natural Language Inference (NLI) and claim-source alignment to assess summary factuality (Scirè et al., 2024). These entailment-driven frameworks offer a promising template for detecting hallucinations in legal AI. However, the lack of domain-specific resources aligned with the precise logic of judicial reasoning remains a significant challenge for widespread adoption. Recent surveys argue for more rigorous evaluation protocols including structured rubrics and long-context tasks to better capture the nuances of legal LLMs (Dehghani et al., 2025; Akter et al., 2026). Audits consistently show that fluent retrieval-augmented outputs often hallucinate authorities, making verification layers mandatory (Magesh et al., 2025). While LLM-based evaluators offer a scalable path for assessment, they carry their own faithfulness risks; a more prudent approach triangulates such scores with source-grounded checks and human review (Dehghani et al., 2025). A practical compromise is to report standard overlap metrics for comparability while adding targeted consistency tests and detailed error analysis (Deroy et al., 2023) (Table 1).

**Table 1:** Overview of methodological families in legal judgment summarization.

Approach	Typical methodology	Illustrative studies	Strengths	Limitations
Feature-based and classical ML	Hand-crafted features, statistical ranking, classifiers for sentence salience	(Kanapala et al., 2019; Bhattacharya et al., 2019)	Strong extractive baselines with modest data needs	Manual feature design; limited modeling of discourse dependencies
Transformer based Deep Learning	Pretrained encoders, long-document variants, abstractive decoders	(Chalkidis et al., 2021; Shukla et al., 2022; Zhong et al., 2020)	Strong contextual representations and fluent outputs on benchmarks	May under-represent clause-level premises; precedent structure often implicit
LLM-era summarization	Prompting, fine-tuning, retrieval augmentation	(Deroy et al., 2023; Jayakumar et al., 2023; Siino et al., 2025)	Flexible generation; long-context handling	Hallucination risk; governance and verification burden
Segmentation and argument mining	Sentence or clause segmentation; rhetorical-role tagging	(Poudyal et al., 2020; Xu and Ashley, 2023; Nigam et al., 2025)	Interpretable units aligned with legal reading	Annotation cost; domain shift across courts
Graph-based and citation-aware models	Graph convolution or attention on sentence or case graphs; citation graphs for extractive summaries	(Yasunaga et al., 2017; Huang et al., 2021; Zhao et al., 2022; Bindal et al., 2023; Dan et al., 2025)	Captures relational and citation topology	Sensitive to graph quality; compute cost at scale
Precedent-aware and inter-document models	Linking citations and related cases	(Huang et al., 2021; Wu et al., 2023)	Brings inter-document authority into the loop	Still few end-to-end summarizers with full precedent reasoning

## 4 Identified research gaps and justification

One limit concerns this article itself. It is a mini review, not a full systematic review. The Introduction describes search and screening, but we do not provide a formal selection-bias or risk-of-bias assessment across included studies. That matches common practice for this article type while keeping the scope transparent.

Despite the progress, several gaps limit practical impact and cross-jurisdiction generalization. Early extractive and thematic systems showed that legal text can be segmented, but often without explicit argumentative roles tied to judicial logic (Grover et al., 2003).

Transformer-based approaches including LegalBERT-related work improve semantic modeling, but still often operate primarily at sentence granularity, missing multiple propositions within one sentence (Chalkidis et al., 2019; Shukla et al., 2022). Argument relations may be under-specified in the summary output.

A significant amount of the literature relies on public datasets that reflect specific legal systems, notably from European or U.S. jurisdictions (Chalkidis et al., 2019; Bench-Capon and Dunne, 2007; Ashley, 2017). Developing methods tightly coupled to a single corpus risks limiting transferability. Consequently, robust legal AI requires a commitment to multilingual and multi-jurisdictional evaluation to ensure broader applicability and generalization (Benedetto et al., 2025).

Precedent citations are still often treated as metadata rather than first-class elements of the reasoning graph that the summary must reflect (Shukla et al., 2022; Zhong et al., 2020).

Despite the high stakes of judicial work, evaluation remains disproportionately centered on lexical overlap metrics. However, high scores in fluency or ROUGE are no guarantee that a summary preserves core legal principles (Lin, 2004; Shukla et al., 2022). There is a clear, yet underdeveloped, need for protocols that integrate automated scores with structured expert review, NLI-based factuality checks, and argument-aware frameworks (Ghosh et al., 2022; Scirè et al., 2024; Akter et al., 2026). Given the risks associated with LLM hallucinations, grounding these models in verifiable legal context is now a primary research priority (Deroy et al., 2023; Dehghani et al., 2025; Magesh et al., 2025).

## 5 Discussion

This review has mapped the evolving landscape of automated legal summarization, highlighting both gains in model performance and persistent limitations of current architectures. While Transformer-based models have raised the bar on standard metrics, many systems still misalign with interpretive workflows in legal practice. Judicial reasoning is not a linear sequence of text but a structured network of arguments embedded in a web of authority and precedent. Sentence segmentation often oversimplifies that multi-role structure; clause-level analysis can recover finer argumentative detail, yet such methods are still seldom folded into unified end-to-end summarizers, which limits their effect on summary quality (Xu and Ashley, 2023; Nigam et al., 2025).

Rhetorical-role labeling improves interpretability, yet many abstractive systems still optimize fluency. Legal summarization is therefore not only a language-generation problem but partly a reasoning and discourse problem (Malik et al., 2022; Shukla et al., 2022). Citations should often be treated as part of the reasoning story, not as peripheral metadata. Graph-based methods are promising but frequently emphasize citation prediction or classification rather than summarization objectives that depend on precedent content (Huang et al., 2021; Wu et al., 2023). ROUGE and BLEU remain convenient but misaligned with legal needs; checklist- and rubric-oriented views in LLM and legal summarization surveys, together with NLI-oriented factuality metrics, reinforce the need for richer evaluation protocols (Dehghani et al., 2025; Akter et al., 2026; Scirè et al., 2024).

In sum, the survey covered theoretical foundations (Section 2), methodological families from classical machine learning to LLMs (Section 3), and explicit gaps (Section 4). The literature suggests a clear evolution from simple extractive methods toward structured, defensible summarization frameworks that combine clause- and role-aware segmentation with graph-based or citation-aware design, and toward measured use of generative models. Where legal stakes are high, systems still need to be anchored by robust retrieval, verification layers, and essential human oversight (Ashley, 2017; Deroy et al., 2023; Nigam et al., 2025).

## 6 Conclusion

In conclusion, the current trajectory of the field suggests that future breakthroughs will rely less on the brute-force scaling of generative architectures and more on a structure-first design philosophy. To truly capture the nuance of judicial reasoning, research must pivot toward finer-grained segmentation, transitioning from sentence-level baselines to clause-level analysis, while integrating precedents and citations directly into the modeling loop. Furthermore, evaluation frameworks must mature beyond the convenience of standard n-gram overlap metrics to address the substantive legal risks inherent in automated summarization. By coupling retrieval-augmented verification with rigorous structural oversight, we move toward a generation of tools that do not merely simulate fluency, but actively track the complex logic of the law.

This mini-review aimed to chart that trajectory from classical extractive work through transformers and LLMs, and to foreground gaps that matter most for judgment-oriented summarization. We hope framing these priorities helps orient follow-on work toward more robust, argument-aware legal AI.
